# Factors Associated with the Uptake of Human Papilloma Virus Vaccine Among Adolescent Girls Aged 14 Old in Hai District of Kilimanjaro Region in Northern Tanzania

**DOI:** 10.24248/eahrj.v8i2.789

**Published:** 2024-06-26

**Authors:** Jojet N Josephat, Russel B Halama, Harieth F Makiriye, Amina Farah, Irene Haule, Margaret Kirumbuyo, Caroline Amour, Sia E. Msuya

**Affiliations:** a Institute of Public Health, Department of Community and Global Health, Kilimanjaro Christian Medical University College (KCMUCo), Moshi, Tanzania; b District Medical Officer, Hai District Council, Kilimanjaro, Tanzania; c Institute of Public Health, Department of Epidemiology & Biostatistics, Kilimanjaro Christian Medical University College (KCMUCo), Moshi, Tanzania; d Department of Community Health, Kilimanjaro Christian Medical Centre (KCMC), Moshi, Tanzania.

## Abstract

**Background:**

Human Papilloma Virus (HPV) vaccination is a key primary prevention method against cervical cancer which is given to young girls before onset of sexual activity. In Tanzania, cervical cancer is the most frequent occurring cancer among women and is the leading cause of cancer mortality. The HPV Vaccination programme was rolled-out in Tanzania in 2018 for adolescent girls aged 9-14 years to receive two doses at a six-months interval, with an annual vaccine uptake of 46.1%. In 2019, the uptake of the HPV vaccine was only 64%, whereas the national coverage target is 75%. This study aimed at assessing the determinants of HPV vaccination among adolescent girls in Hai district, Kilimanjaro region, northern Tanzania.

**Methodology:**

A cross-sectional study was conducted from July 2020 to August 2020 among 14-year-old adolescent girls in four selected secondary schools, including two government-owned and two private-owned, within Hai district. Close-ended questionnaire interviews were conducted with the adolescent girls in the study and data analysis was done using IBM SPSS Statistics for Windows version 20.0 (IBM Corp, Armonk, NY, USA). Odds ratio was used to assess the association between several factors and HPV vaccination.

**Results:**

A total of 301 adolescent girls aged 14 years consented to the study. HPV vaccination uptake was 65%. More than three-fifths (n=119, 60.7%) reported having received the two required doses. Knowledge of HPV (OR 5.68; 95% CI, 0.72 to 44.96; *P=.01*) and HPV vaccine (OR 20.11; 95% CI, 10.88 to 37.99); *P =.01*) contributed significantly to HPV vaccine uptake among the adolescent girls in the study. More than one-third (n=105, 34.9%,) of the participants, were not vaccinated, the main reasons adduced for not being vaccinated include lack of proper information about the HPV vaccine (60.0%), fear of side effects (14%) and parental refusal (11%).

**Conclusion:**

HPV vaccination uptake was 65%. Lack of proper information to both the children and parents about the safety of the vaccine hinders its uptake. More effort should be made for clear and comprehensible dissemination of information especially to the community stakeholders mainly parents, community and religious leaders, about cervical cancer and HPV vaccine in order to considerably increase vaccination coverage among adolescent girls. Likewise, involvement of healthcare workers and policymakers in educating people about cervical cancer prevention measures can ensure successful implementation of HPV vaccination. There is need to conduct an indepth qualitative study to explore further people's perceptions and attitudes towards HPV.

## BACKGROUND

Human Papilloma Virus (HPV) is a DNA virus that infects the skin or mucosal cells. It is the most leading cause of cervical cancer.^1^ There are more than 100 HPV genotypes where fourteen genotypes are internationally classified as “high risk” including HPV 16 and 18 which account for 70% of cervical cancer cases.^1^ The virus is most commonly found on skin around the genitals. It is transmitted via skin contact most commonly through sexual intercourse and men are the main carriers.^1^ HPV infections can take more than 10 years to develop cervical cancer. Risk factors of cervical cancer caused by HPV include multiple sexual partners, and immune-compromised diseases and disorders such as HIV/AIDS victims.^2^

Cervical cancer is the fourth most frequent cancer in the world among women with an estimate of 570,000 new cases in 2018 and approximately 311,000 deaths covering 6.6% of all female cancers.^3,4^ Approximately 88% of deaths from cervical cancers occurred in low and middle income countries (LMIC).^1^ In Sub-Saharan Africa, cervical cancer is the second leading female cancer after breast cancer with estimates of 93,225 new cases and 57,381 deaths.^5^ In Tanzania, cervical cancer is the most occurring cancer among women. Statistics from the Ocean Road Cancer Institute (ORCI) hospital shows that cervical cancer patients account for 36% of all cancer patients and about 80% of them die within five years of diagnosis.^2^

The significantly reduced occurrences of cervical cancer globally, has been attributed to utilization of cervical cancer prevention strategies.^6^ There are secondary and primary prevention strategies including cervical screening methods with high-positive predictive value such as HPV DNA testing among sexually active women and treatment of pre-cancerous lesions. The primary prevention such as HPV vaccination has been used among young girls especially those that are not sexually active.^6,7^ to verify whether or not the high efficacy reported in randomised controlled clinical trials are materialising in real-world situations. Methods: We searched the Medline and Embase databases (between Jan 1, 2007 and Feb 28, 2014 HPV vaccination has been predicted to reduce about 89% of cervical cancer incidences in low and middle-income countries and avoid over 60 million cases within the century.^8^ The enrolment of the HPV vaccine started universally in 2014, in most of the high-income countries. The vaccine uptake was generally low and varied with most them having achieved an average of less than 50%.^5^ By 2016 with the support of the Global Alliance for Vaccines and Immunisation (GAVI), around 47 LMIC introduced the vaccine to their national programmes, leaving out most of LMIC with higher incidence risk for cervical cancer.^9^

Tanzania has adopted both primary and secondary prevention strategies to decrease cervical cancer incidence, morbidity and mortality. In 2011, a country scaled-up cervical cancer screening programme was conducted using Visual Inspection with Acetic Acid, and treatment of positive cases using cryotherapy or LEEP.^2^ In 2014, HPV vaccine was first piloted in Kilimanjaro region, Tanzania. It involved vaccine administration of two doses of quadrivalent Gardasil HPV vaccine to school girls in and out of school girls aged 9-14 years and achieved an overall coverage of 92.8%. In 2018 the HPV vaccination programme was nationally introduced, including Gardasil HPV vaccine given in two doses at an interval of six months. The vaccine was to be given to the upper age cohort of girls at 14 years and there were preparations to extend the programme to the rest of the eligible age (9-14 years) by 2020, with national uptake target of 75%.^2^

After the national roll-out programme, 26 out of 32 regions successfully adopted the vaccination programme. The HPV vaccine uptake was 46.1% with 59.9% for HPV-1 and 32.4% for HPV-2. In 2019 the coverage performance was 63.5% with 78% for the HPV-1 and 49% for HPV-2.^2^ The average uptake of HPV vaccine among adolescent girls in Tanzania from 2018 and 2019 is 56.3% which is below the national target of 75% and the uptake of the second dose is below 50%. In 2019, Kilimanjaro region had HPV vaccine coverage of 35.5% with Hai district having the lowest HPV vaccination coverage of 14% among the 7 districts in Kilimanjaro Region. The reasons for such low uptake were unclear. This study, therefore, aimed at assessing the determinants of HPV vaccine uptake among adolescent girls in Hai District, Kilimanjaro Region, northern Tanzania.

## METHODS

### Study Design and Site

This was a cross-sectional study conducted from July 2020 to August 2020 among adolescent girls aged 14 years old in four secondary schools in Hai district located in Kilimanjaro region, which is located in the northeastern part of Tanzania. Kilimanjaro region is made up of seven districts namely Same, Mwanga, Rombo, Moshi urban and rural, Siha and Hai districts. Hai district is administratively sub-divided into 10 wards with a total population of 210,533 according to the 2012 Tanzania National Census (Census, 2012). There are 47 secondary schools, 29 government owned and 18 private owned. According to Vehicle Health Monitoring System (VHMS) information on national vaccine coverage by June 2020, Kilimanjaro region had HPV vaccine coverage of 35.5% with Hai district having the lowest HPV vaccination coverage of 14% among the 7 districts in Kilimanjaro region.

### Study Population and Sampling; Population

The study population included adolescent girls in form one and two classes within four selected secondary schools in Hai district, who reported to be 14 years of age and gave verbal assent to participate in this study. The study excluded students who were not at school on the day of data collection because it was an in-school study.

### Sample size

Sample size for this study was estimated by using the Cochran's formula for for sample size determination,^10^



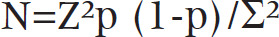



Where N is the estimated minimum sample size, Z is the confidence level at 95% (where the standard value is at 1.96), P is prevalence, we used 63.5% which was the prevalence of HPV vaccination among adolescent girls in 2019. and Σ= Margin error (the error that can be tolerated=5%). The minimum sample that was required for this study was 356 adolescent girls aged 14 years old. The addition of 5% for non-response gave a minimum sample of 374 adolescent girls aged 14 years old in Hai district by 2020.

### Sampling

Hai district council was selected due to its average low uptake of HPV vaccine among adolescents aged 14 years in 2020, among the seven districts in Kilimanjaro Region. Four secondary schools, including Boma secondary and Hai secondary which are government owned and Shilela secondary school and St Dorcas secondary school which are private owned, were randomly selected from a pool of secondary schools in Hai district. The names of these secondary schools were written on small pieces of paper and placed in a non-transparent bag, then two government-owned and two private owned schools were randomly selected. Secondary school girls in form one and form two class level were purposely selected as majority of them at this education level are aged 14 years. The total number of adolescent girls in the given class level from these four secondary schools was 500 students. About 301 adolescent girls aged 14 years old from these classes who verbally assented to the study were enrolled. At least 85 adolescent girls aged 14 years old were required to be interviewed from each of the four secondary schools and were randomly selected.

### Data Collection Method, Tools and Procedures

Interviewer-administered questionnaire was used to collect key information from the participants using. Three 4^th^ year medical undergraduate students who were trained collected the data from the study participants. The questionnaire was pre-tested on 10 girls in form two aged 14 years old at one of the secondary schools in Moshi urban district in Kilimanjaro region to check for reliability, consistency, clarity, and flow of the questions. The reliability by Cronbach Alpha value of 0.8.

The questionnaire used contained closed-ended questions divided in three sections addressing socio-demographic information, HPV uptake levels, and factors affecting the uptake. Information on reasons for failure to vaccinate for those who had not been vaccinated was also collected in the third section.

After permission was obtained to conduct the research, data was collected with the consent and assistance of the school administration. Assent obtained from each study participant. The Survey Chief Technology Officer (CTO) data collecting tool containing English and Swahili translation of questions about HPV vaccine uptake and factors associated with the uptake of HPV vaccine was used to collect data. The interview was conducted after class hours in a prepared classroom and took about 20-25 minutes per participant. Due to COVID-19 pandemic, we ensured all interviews were conducted with social distancing, wearing face masks, and proper hand washing with soap and running water or sanitizers.

### Data Analysis

Data was transferred from survey-CTO into SPSS software. Descriptive analysis was used to summarize data. Continuous variables data were summarized using mean or median with variance as measures of dispersion. Categorical variables were summarized by frequencies and percentages. Bivariate analysis used to test associations was done using Fisher's Exact Test for categorical variables and *P* value of <0.05 was taken for statistically significant results. Multivariable logistic regression analysis was carried to control for confounders and identify independent predictors for HPV-vaccination uptake.

### Categorisation of Variables

Uptake of HPV vaccine was assessed by asking all the study participants if they had ever received the HPV vaccine or not. Knowledge of HPV was measured by asking 9 questions on general knowledge and or awareness of HPV involving how it is transmitted, its effects, how it can be diagnosed and prevention methods such as condom use. Knowledge about HPV vaccine was measured by asking 11 questions that included the vaccine frequency and intervals, where and who can administer it, and at what age, its benefits and side effects. The outcome variables were binary into Yes =1 and No =2. Participants with up to 5 correct responses about HPV and about HPV vaccine, were categorized as having adequate knowledge of HPV and HPV vaccine.

### Ethical Consideration

The study received ethical approval from the Kilimanjaro Christian Medical University College (KCMUCo) Research Ethics Committee. The permission to conduct the study was obtained from the Hai district council authority and the office of District Executive Director. Consent was sought from the heads of the schools as guardians for the adolescents while at school and verbal assent was sought from the adolescent girls before participating in the study. We used identification numbers for the participants instead of their names, to maintain anonymity.

## RESULTS

### Socio-Demographic Characteristics of the Participants

Three hundred and one (301) adolescent girls aged 14 years old, 80% of the expected sample size, consented and were enrolled in the study, with 79% of them from government-owned and 21% from private-owned secondary schools. Sixty-six percent of the girls were in form one and about 234 (77.7%) students live in urban areas, Majority, 211 (70.0%) had caretakers with secondary education level. Nearly 94% of the participants reported not having boyfriends (Table 1).

**Table 1.** Background Characteristics of the Participants (N=301)

Characteristics	Frequency	Percentage (%)
Residence		
Urban	234	77.7
Rural	67	22.3
Religion		
Muslim	115	38.2
Christian	186	61.8
Class level		
Form 1	200	66.4
Form 2	101	33.6
Head of household		
Father	39	13.0
Mother	1	0.3
Grandparents	240	79.7
Others	21	7.0
Caretaker level of education		
Informal education	20	6.6
Primary education	70	23.3
Secondary education	128	42.5
Higher education	83	27.6
Boyfriend		
Yes	17	5.6
No	284	94.4
Number of people in the household		
1-5	191	63.5
6-10	104	34.6
10+	6	1.9
Family ownership (“Yes” response, each out of 301)		
Radio	270	89.7
Television	258	85.7
Internet access	237	78.7

### Uptake of HPV Vaccination

Of the 301 adolescent girls enrolled in the study, 196 (65.1%) reported to have received at least one dose. Of those vaccinated (196) 119 (60.7%) students reported to have received both doses of the HPV vaccine. Taking the total enrolled students as a denominator, the HPV-1 coverage was 65% and the HPV-2 was 39.5%. Of the 105 (34.9%) who were not vaccinated, 47 (44.8%) agreed to get vaccinated if there was an opportunity, (Table 2).

**Table 2.** Uptake of Human Papilloma Virus Vaccine Among Girls in Hai District (N=301)

Variables	Frequency	Percentage (%)
Received HPV vaccine		
Yes	196	65.1
No	105	34.9
If answered YES, how many doses did you receive (n=196)		
One dose	71	36.2
Two doses	119	60.7
I don't remember	6	3.1
If answered NO, would you want to get vaccinated? (n=105)		
Yes	47	44.8
No	21	20.0
I don't know	37	35.2

### Association Between Socio-Demographic and Socio-Economic Factors and HPV vaccination

The association between socio-demographic and other factors with uptake of HPV vaccination among adolescent girls is shown on Table 3. HPV vaccine uptake was associated with having good knowledge of HPV and HPV vaccine as well as owning a radio as a means of access to information. Adolescents with knowledge of HPV vaccine had 20 times higher odds of uptake HPV vaccine than others. The adolescent girls from families that owned a radio as a means of information were twice as likely to receive the HPV vaccine as the families with no radio ownership.

**Table 3.** Association Between Socio-Demographic, Socio-Economic and Knowledge of HPV and HPV Vaccine Uptake Among Adolescents in Hai District (N=301)

Variable	HPV Vaccination			Adjusted OR 95% CI	P Value
	Yes	No	OR		
Religion					
Muslim	72	43	Ref		
Christian	124	62	1.19	0.67-1.79	.001
Head of household					
Father/mother	26	13	Ref		
Grandparents	151	89	0.84	0.58-2.44	.134
Others	19	2	4.75	1.29 – 24.88	.050
Head of household education level					
Higher education	55	28	1.96	0.66-2.15	.002
Secondary education	79	49	1.61	0.33-1.36	.050
Primary education	52	18	2.88	0.71-5.19	.226
Informal education	10	10	Ref		
Source of information about HPV vaccine					
Internet access	147	90	Ref		
Radio	179	91	1.97	0.90-4.48	.234
Television	166	92	1.80	0.38-1.70	.061
Knowledge of HPV					
Yes	11	0	1.63	0.25-0.92	.038
No	185	105	Ref		
Knowledge of HPV vaccine					
Yes	160	19	20.1	10.88-37.99	.031
No	36	86	Ref		

Abbreviations: HPV, Human Papilloma Virus

### Individual and Community Reasons for Failure to Uptake HPV Vaccination

**Figure 1.**
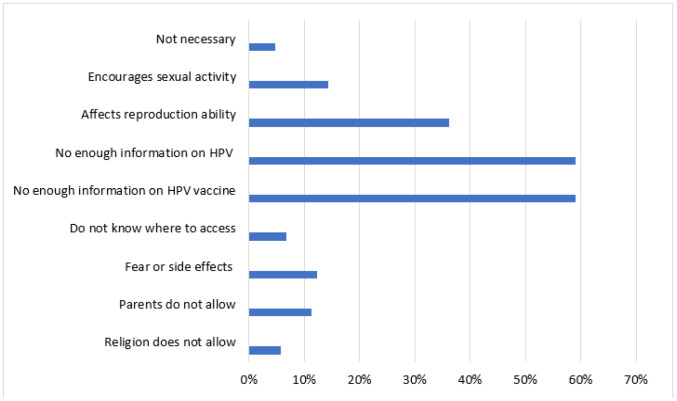
Reasons for not Receiving Human Papilloma Virus Vaccine Among Adolescent Girls in Hai District

About 35% of the adolescent girls in the study did not receive the HPV vaccine due to several individual and community reasons as shown well in Figure 1. Individual reasons included not having enough information about the HPV and HPV vaccine (59%) and fear of its negative outcomes on the reproductive organs (36%). Among community reasons for not getting vaccinated included parental refusal (11.4%) and religious prohibition (5.7%).

## DISCUSSION

In this cross-sectional analytical study, we assessed the uptake of the HPV vaccine and factors that hindered HPV vaccination among adolescent girls aged 14 years who were in secondary schools in Hai district, Kilimanjaro region in 2020.

The results of this study showed that the proportion of adolescent girls who received the HPV vaccine was 65%. Out of these girls who received the vaccine, 60.7% received the two recommended doses and about 40% missed the second dose. The study also revealed that adequate knowledge of HPV had about 90% influence on HPV vaccination. Of the 105 adolescent girls who did not receive the vaccine, 47 (44.8%) of them wanted to be vaccinated if given opportunity.

The 35% of the girls who were not vaccinated presented various reasons for that, mainly lack of access to proper information about HPV and HPV vaccine including the importance of being vaccinated.^11^ More than 90% of the girls who were not vaccinated had no information and wished to know more about HPV and the HPV vaccine. This finding corresponds to study results from China, Nigeria, Kenya, and Tanzania.^5,12,13,14^

Parental disapproval accounted for 11.4% of adolescent girls missing HPV vaccination. Among the opinions given by parents about the HPV vaccine involved included encouragement of sexual activities at a young age which is against their religious and cultural norms.^15,16^ The adolescent girls in this study who reported living with other relatives as their heads of household accounted for 90% of HPV vaccination uptake with four times more odds of being vaccinated than the ones having parents as their heads of household. Lower acceptance of the vaccine in East Africa has been associated with parents as family decision-makers due to parental refusal of the vaccine.^17,18^

About 3 in 10 adolescent girls from this study who did not receive the vaccine reported fear that the HPV vaccine may cause infertility. Fear of negative medical implications of the vaccine is one of the major barriers to the HPV vaccine uptake in Sub-Saharan Africa.^17^

Increased efforts to provide appropriate education to adolescent girls and their guardians are required to improve the uptake of HPV vaccination.^19^ Free enrolment of the vaccine programme nationally and facilitating education about HPV and HPV vaccine to the stakeholders were among the GAVI alliance requirements to ensure mass awareness and encourage HPV vaccine uptake.^20^

There is a need to design a simple educative program on HPV and HPV vaccine that targets adolescent girls from 9 years old, their primary care takers at home and at school and health care providers, which provides elaborate and clearly disseminated information to prevent misinformation ^2^

It is important to develop a comprehensive educational programme about HPV and HPV vaccine, targeting adolescent girls from age 9 and their primary caregivers at home and schools. This programme should be delivered by healthcare providers to ensure its effectiveness and reach.^2 21^

The main strength of this study was the use of quantitative analysis of the data collected using questionnaires that gave baseline information on proportion and factors associated with the uptake of HPV vaccine among adolescent girls, hence laying a foundation for making proper policies and strategies in the enrolment of the HPV vaccine programme to reach the national target.

The main limitations of this study include COVID-19 related restrictions; we were not able to achieve the minimum sample calculated. Also limited community access due to the COVID-19 effect, the study was conducted in two out of the ten wards in Hai district. Although there are no discernible differences in socio-economic levels, urbanity, ethnicity, and school distribution in other wards, collecting data from selected schools across the entire district may have provided a more complete picture of the proportion of adolescent girls vaccinated as well as barriers to vaccine uptake. There is also a possibility of social desirability bias among adolescent girls enrolled in the study where they may have over-reported having been vaccinated as it would reflect well on them. Additionally, we did not investigate the reasons why the unvaccinated adolescent girls were uncertain about receiving the vaccine which potentially limits our understanding of their vaccination preferences.

## CONCLUSION

The HPV-vaccine uptake is low with more than a third (35%) of the eligible girls for HPV vaccination missing the vaccine altogether and about 40% of the girls vaccinated with the first dose are lost to the second vaccination. Poor knowledge of HPV and HPV vaccine, fear of side effects among adolescent girls, parental refusal and religion beliefs were among the reasons for the low uptake of HPV vaccine.

We recommend conducting massive HPV community awareness campaigns for sensitization, involving the major stakeholders such as adolescent girls, parents and teachers. This could be achieved through targeting audiences in social and religious gatherings. Appropriate and extensive use of social media innovations to reach out to the adolescents provides a reliable platform given that 8 in 10 adolescents have access to the internet and information mediums such as radio and television. There should be increases use of mass media to disseminate comprehensible information about HPV, HPV vaccine and cervical cancer to society and facilitate its successful implementation.
